# Learning from volcanic eruptions: Co-production of knowledge at Merapi and Kelud, Indonesia

**DOI:** 10.4102/jamba.v17i1.1881

**Published:** 2025-08-26

**Authors:** Nuzul Solekhah, Fatwa N. Hakim, Eko Wahyono, Reza A. Prayoga, Siti Fatimah, Lis Purbandini, Djoko P. Wibowo, Rachmini Saparita, Febby Febriyandi YS, Muhammad Alie Humaedi

**Affiliations:** 1 Research Center for Social Welfare, Village and Connectivity, National Research and Innovation Agency, Jakarta, Indonesia; 2 Department of Anthropology, Gadjah Mada University, Yogyakarta, Indonesia; 3 Department of Sociology, Faculty of Social and Political Sciences, University of Indonesia, Depok, Indonesia; 4 Research Center for Environmental Archaeology, Maritime Archaeology, and Cultural Sustainability, National Research and Innovation Agency, Jakarta, Indonesia

**Keywords:** building trust, co-production, knowledge, mitigation, post-eruption, volcano

## Abstract

The increasing frequency and intensity of volcanic disasters present major challenges for rural communities, particularly in terms of recovery and the restoration of environmental conditions post-crisis. Despite extensive disaster management literature, gaps remain in understanding the social dynamics and historical experiences of rural populations affected by volcanic eruptions. This study investigates the role of co-production as a mitigation strategy to enhance community resilience and improve disaster response in volcano-prone rural areas. The research focuses on two rural villages in Indonesia: Pandansari, located on the slopes of Mount Kelud in Malang Regency, and Kemiren, situated on the slopes of Mount Merapi in Magelang Regency. A qualitative research paradigm was employed, using a life history approach to collect in-depth narratives from community members. Sixteen participants were purposively selected across both villages. Data were gathered through semi-structured interviews and focus group discussions, followed by thematic analysis to identify key patterns and themes. Findings indicate that local knowledge systems play a critical role in shaping community behaviours and responses to volcanic disasters. These systems enable communities to mobilise public goods and restore livelihoods in ways that reflect pre-disaster norms and conditions. Integrating co-production strategies into disaster management practices can significantly improve community resilience and the effectiveness of response mechanisms.

**Contribution:**

This study advances the understanding of how historical context and local knowledge inform disaster responses in rural settings, offering practical implications for participatory and community-based disaster risk reduction.

## Introduction

Indonesia is a tropical climate region comprising around 17 000 islands, with 922 of the islands permanently inhabited (Pambudi 2018). Beneath its rich natural diversity, Indonesia lies at the convergence of three major tectonic plates: the Eurasian Plate, the Indo-Australian Plate and the Pacific Plate (Kaban et al. 2019). The interaction of these tectonic boundaries forms part of the Pacific Ring of Fire, a seismically active zone stretching from Southeast Australia to the western coasts of the Americas (Andreastuti, Paripurno & Gunawan 2019; Pambudi 2018). As a result, Indonesia faces heightened vulnerability to natural disasters such as earthquakes, volcanic eruptions and tsunamis (Siagian 2014). In the context of volcanic activity, Indonesia employs a four-level alert system comprising: Level I (Normal), Level II (Alert), Level III (Standby) and Level IV (Warning). At each level, the implementation of mitigation policies, strategies and actions involves not only government intervention but also relies heavily on the willingness of the community to cooperate (Prayoga et al. 2024; Saparita et al. 2024).

The characteristics and behaviour of communities residing in a particular area often result in differing responses compared to those in other regions (Bevilacqua et al. 2023; Bird, Gísladóttir & Dominey-Howes 2011). For instance, the eruptions of Mount Kelud in 2007 and 2014 exhibited distinct characteristics. The 2007 eruption was effusive, resulting in the formation of a crater (Bélizal 2012), whereas the 2014 eruption, although it caused no fatalities, was a Plinian-type event that destroyed the summit of the volcano (Nakamichi et al. 2019; Sudarmanto 2020). The eruption of Mount Kelud is typically brief and high-velocity (Bachri 2021). Because of its proximity to Mount Kelud and the typically short-lived nature of eruptions, residents of Pandansari village generally do not have secondary housing elsewhere. To date, villagers in Pandansari have relied on local knowledge and collective memory to respond to eruptions of Mount Kelud. According to their lived experiences, before 2014, volcanic material almost always flowed towards Blitar and Kediri. Many believe that Mount Amping, which is higher than Mount Kelud, acts as a natural fort when the volcano erupts. This belief reinforces a local myth that Pandansari village is safer than the surrounding areas. As such, residents willingly engage in collaborative mitigation efforts led by the village government, the Provincial Disaster Management Agency (BPBD), the Indonesian National Armed Forces (TNI) and the Indonesian National Police (POLRI). The community participates in public awareness campaigns and evacuation drills, yet maintains confidence in the assumption that the village remains as safe as in previous years, despite ongoing challenges related to evacuation, particularly where socio-cultural norms are concerned (Gabrielsen et al. 2017; Purworini, Hartuti & Purnamasari 2021). Since the 2014 eruption, the collective memory of that experience has fostered a more cooperative attitude towards disaster mitigation among the residents.

In contrast, Mount Merapi is characterised by a more dynamic and fluctuating eruption pattern (Holmberg 2023). Among Indonesia’s 130 active volcanoes, Merapi is one of the most active, with a pattern of recurring effusive and explosive eruptions that occur on a 4-year cycle (Hastuti 2019). Its 2010 eruption was particularly devastating and resulted in 339 fatalities (Hastuti 2019). In the aftermath of this disaster, recovery and rehabilitation efforts, including river normalisation to manage lahars and volcanic debris, altered the physical landscape. These changes, in turn, created opportunities for land-use shifts, particularly in the form of renewed and intensified sand mining. One of the communities most affected by this shift is Kemiren village, which has struggled to stem the growing prevalence of large-scale sand mining practices to this day. Villages that permit sand mining around Mount Merapi view it as an economic opportunity. Meanwhile, those who oppose it raise concerns about the impact of heavy truck traffic on evacuation routes (Mei et al. 2013).

These two different situations illustrate the current trajectory of disaster experiences among residents living in the vicinity of Mount Merapi and Mount Kelud. While the definition of disaster has been the subject of considerable academic discourse, what remains crucial is understanding what renders specific communities and locations vulnerable or resilient to environmental hazards and unforeseen events (Furedi 2007; eds. Perry & Quarantelli 2005). In some literature, vulnerability is often defined as a condition of limited capacity to cope with exposure to environmental and social stresses, alongside an absence of adaptive mechanisms (Kasperson et al. 2022).

Numerous studies have explored vulnerability in the areas surrounding Mount Merapi and Mount Kelud from multiple perspectives. For example, Hardiansyah et al. (2020) examined the vulnerability of road networks and evacuation routes in the Merapi area. Hisbaron (2018) highlighted the application of Spatial Multi-Criteria Evaluation (SMCE) as a tool to capture the intersection of physical, social and economic characteristics, and to identify determinants of community resilience. Additional research has explored social vulnerability in volcanic regions, focusing on key factors and policy implications (Siagian 2014). Other studies have employed computational analysis and technical mapping such as physical building damage vulnerability analysis and geographic analysis (Maharani et al. 2020; Williams 2020). While these studies provide valuable structural and spatial insights, this research offers a complementary perspective by exploring how residents’ understanding of vulnerability has evolved, using a life history approach.

The research conducted by Glass and Davis (2004) offers a critical deconstruction of vulnerability within the framework of postmodern feminist inquiry, arguing that vulnerability does not always signify a negative condition. This study draws on Glass and Davis’ (2004) conceptualisation, in which emotional, cognitive and behavioural aspects associated with vulnerability can be transformed into a foundation for positive agency in confronting helplessness and threats. In line with the deconstruction approach, this study aims to examine how lived experiences and socio-ecological conditions in disaster-prone areas influence the co-production of knowledge, specifically how residents reinterpret vulnerability in ways that inform mitigation strategies and reshape socioeconomic relations. Through a life history approach, the study investigates the historical context of how residents perceive volcanic disasters, with a focus on the evolving forms of resilience and collective action following the eruption of Mount Merapi and Kelud.

Building on this inquiry into the lived meaning of vulnerability, the research is grounded in the conceptual framework of *Co-Production of Knowledge*, particularly in relation to social change, adaptive strategies and community-based ecological responses. In this context, co-production refers to the integration of socially derived knowledge, rooted in collective empirical experience, cultural practices and environmental interactions. It is understood as a collaborative process of knowledge creation that emerges through interaction among community actors (Howarth & Monasterolo 2017). Successful co-production relies on key elements, including inclusive participation, a shared vision, effective leadership, shared responsibility and open communication (Hegger & Dieperink 2014).

This study focuses on communities in the Mount Merapi and Mount Kelud regions, where co-produced knowledge has emerged organically, without direct state intervention, as a form of bottom-up resilience. The harsh ecological conditions of these volcanic zones compel residents to develop new social practices and locally grounded responses. This contrasts with previous studies that examined co-production in formal or institutional settings, highlighting instead a more spontaneous and community-led process of knowledge creation (Redman et al. 2021; Zurba et al. 2022).

## Research methods and design

### Research approach

To address the research problem, this study adopts a life history approach. Initially developed within the field of anthropology and later adopted by sociology (Goodson 2001), the life history method has been critiqued by positivist scholars but remains valuable for its ability to capture experiences through an authentic lens and to amplify the voices and knowledge of the individuals studied (Wright 2019). Within this perspective, life history is not solely concerned with the narrative of personal experience but also with the contextualisation of that experience in relation to social, cultural and material conditions, as well as the individual’s positioning within these structures as they navigate their present and plan their future (Gordon & Lahelma 2003).

The life story approach, as understood from the perspectives of communities living near Mount Kelud and Mount Merapi, serves as a key strategy within this qualitative research. This approach was deliberately chosen to enable a deeper exploration of participants’ experiences and knowledge in a manner that is holistic, systematic, accurate and grounded in empirical reality, particularly concerning the knowledge co-production as a social phenomenon in disaster-prone areas. The philosophical foundation of the qualitative methodology in this study is rooted in the constructivist paradigm, as articulated by Guba and Lincoln (2005), which guides the researcher in uncovering life story narratives as socially constructed realities shaped by co-produced knowledge. Qualitative data were collected through in-depth interviews, observations, focus group discussions (FGDs) and secondary sources, including village archives, video recordings and residents’ documentation captured during eruption events.

### Data collection and analysis

The participants in this study comprised 16 individuals from communities living in the Mount Merapi and Mount Kelud areas. They were purposefully selected based on their experience with shared knowledge related to disaster response and the management of ecological conditions following eruptions. These included survivors of the Mount Merapi and Kelud eruptions, as well as farmers, livestock breeders, volunteers and village officials who were active during the time of the eruptions. Additional interviews were conducted with representatives from the BPBD in Malang and Magelang districts to gather information on the nature and characteristics of volcanic activity in both regions.

Field data collection took place between March and July 2023. To capture residents’ collective memory of eruption events, the researchers conducted FGDs with both individuals and heads of local groups or community organisations. To supplement the diachronic data that could not be accessed in real-time, secondary information was obtained from village government archives. The findings from interviews and discussions were documented through recordings and transcripts, with all data collection activities conducted in accordance with established ethical research standards and with the participants’ approval. The resulting transcripts were then categorised and analysed using a framework guided by the concept of co-production.

### Data analysis

Data analysis was conducted following the interactive analysis model (Miles, Huberman & Saldaña 2018). This model involves several stages: data reduction, data display and conclusion drawing, which are carried out before, during and after the research process. We store the data in a shared storage system and ensure that the confidentiality of both the data and the informants is strictly protected.

### Ethical considerations

Ethical approval to conduct this study was obtained from Badan Riset Dan Inovasi Nasional, Social Studies and Humanities National Research and Innovation Agency Ethics Committee (No. 036 /KE.01/SK/01/2023).

## Result and discussion

### Deconstructing vulnerability

To understand vulnerability, it is essential to clearly distinguish between disasters and hazards (Suyadnya, Novenanto & Tirtayani 2022). Disasters arise from human interactions with the environment and their associated cultural constructs, which are shaped by various identities and span a spectrum from pure objects (nature) to pure subjects (social discourse) (Oliver-Smith & Hoffman 2019). Some scholars interpret vulnerability through the lens of hazard impacts, emphasising its socially constructed nature, as well as the political, economic and exclusionary dynamics that contribute to disaster production (eds. Bankoff, Frerks & Hilhorst 2013). Vulnerability is defined as the degree to which a community is susceptible to disasters in relation to its capacity for resilience (Gillespie & Kofi 2010). Since the turn of the millennium, the focus on vulnerability has increasingly been supplanted by the concept of resilience, which is seen to reflect community agency and adaptive capacity, qualities that contrast with the perceived passivity and negativity often associated with vulnerability (eds. Bankoff et al. 2013).

The United Nations Office for Disaster Risk Reduction (UNDRR) defines resistance as the ability of a system, community or society to withstand, absorb, accommodate, adapt, transform and recover from the impact of hazards within an appropriate timeframe and situation (eds. Bankoff et al. 2013). Although the concept of resilience highlights agency and the capacity to recover, it has also been criticised for obscuring underlying social inequalities and for potentially absolving the state of its responsibilities in ensuring public welfare (eds. Bankoff et al. 2013). This article aligns with Bankoff et al.’s argument that vulnerability remains a critical area of study, as it foregrounds the political, social, economic and environmental processes that expose people to risk (Wisner et al. 2004). While disasters may not be entirely preventable, reducing vulnerability is both possible and necessary. Given that vulnerability is dynamic and subject to change, it must be continuously reassessed (Baycan & Öner 2023; Colvin et al. 2023).

Vulnerability has evolved into a concept that extends beyond its traditional use in disaster mitigation, preparedness, response and recovery. It is now applied more broadly in the study of environmental sustainability, terrorism and social development (Zakour & Gillespie 2013). This article focuses on the concept of disaster vulnerability, which includes susceptibility, risk, resilience and resistance (Zakour & Gillespie 2013). Susceptibility refers to the likelihood of individuals or communities incurring losses as a result of a disaster. Closely related, risk involves the potential for damage, particularly to the physical structures and assets within a community. Resilience refers to the capacity of a human-managed system to recover and return to its original function following a disaster (Adger 2000; Prayoga et al. 2024). Resistance, on the other hand, concerns the strength and durability of physical infrastructure in withstanding disaster-related hazards, such as the ability of bridges, buildings and roads to endure the impact of a volcanic eruption.

To date, vulnerability research has primarily focused on the risks and losses associated with disasters, encompassing both physical damage and impacts on community assets. However, the socio-cultural context of a community influences how strategies to reduce vulnerability are formed (Hettige 2023; Nopriyasman et al. 2024). This article attempts to draw a common thread on how knowledge of the collective memory of volcanic disasters impacts the dynamics of vulnerability in disaster-prone areas. Kemiren is a long-established settlement accustomed to managing the material consequences of Mount Merapi’s eruptions. In contrast, Pandansari village is a relatively new area that experienced severe impacts during the 2014 eruption. These contrasting experiences inform each community’s interpretation of what it means to live in harmony with disaster. As one informant from Kemiren village expressed:

‘It’ s still okay, it’s just raining ash.’ (Interview with Mr. Pur, Age 55 years old, Kemiren village, 07 July 2023)

This is also reinforced by other informants, namely:

‘In the past, if they were told to evacuate immediately, the people who lived here did not believe it, now if they hear the slightest rumbling sound, they are already afraid after 2014.’ (Interview with Mr. Parno, Age 58 years old, Pandansari village, 07 July 2023)

The preceding two responses illustrate a clear contrast in how communities react to volcanic activity. One informant from Kemiren described being familiar with the natural signs that typically precede an eruption. They can categorise these signs as normal, moderate or requiring immediate evacuation. Their knowledge of these eruption patterns reflects a process of normalising the disaster cycle. In contrast, responses of informants from Pandansari village demonstrate a transformation in how the community interprets natural signals from Mount Kelud. The direct impact of the 2014 eruption disrupted the collective belief in the myth of Mount Amping, which was thought to protect the village from volcanic material.

This event marked a turning point for the Pandansari community in understanding and recognising increasingly dynamic natural phenomena as a consequence of living alongside disaster. Previously, residents relied on the belief that Mount Amping would shield them. However, the eruption’s direct impact shattered this conviction, prompting the community to become more alert and responsive to environmental cues. As a result, the people of Pandansari now recognise the importance of more profound knowledge of natural warning signs to support more effective and adaptive mitigation strategies in the face of future disaster risks. Knowledge structures, therefore, play a critical role in shaping the social actions of a community. The Kemiren community’s normalisation of the disaster cycle contrasts with the Pandansari community’s shift away from the geomythology surrounding Mount Amping. These differing frameworks have consequences for local livelihoods.

In Kemiren, the recurring eruptions of Mount Merapi are associated with an abundance of volcanic sand, which has drawn younger residents away from agriculture and into sand mining. This shift has occurred despite the region’s highly suitable soil for cultivating high-quality *salak* (snake fruit). Elders in the community have expressed concern over this trend, fearing that excessive mining will lead to declining groundwater levels during the dry season, reduced spring flow and a gradual loss of soil infiltration capacity, potentially resulting in future droughts.

This situation illustrates that vulnerability in disaster-prone areas is shaped not only by exposure to natural hazards but also by the influence of community knowledge on environmental management. Over time, these dynamics may give rise to new forms of vulnerability at the socio-economic level. One FGD participant shared a personal reflection, noting that he and his son, despite living under the same roof, hold different views on livelihoods. While the father tried to transmit his agricultural knowledge, the son was drawn to mining because of its promise of quicker financial returns. As one FGD participant from Kemiren village stated:

‘Despite living together, my son and I have different views on livelihoods. However, my son is more interested in becoming a miner than a farmer because the income is more promising.’ (Interview with Mr. Janu, Age 52 years old, Kemiren village, 02 July 2023)

**FIGURE 1:**
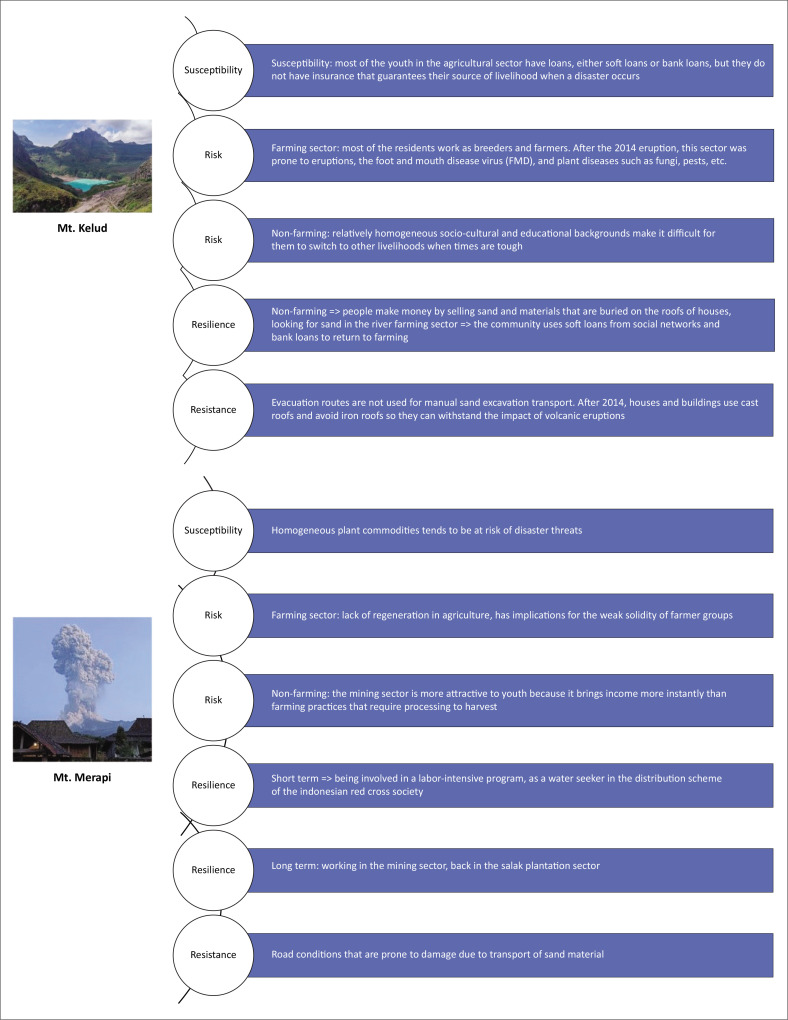
Vulnerability scheme in an eruption-prone community in Mt. Kelud and Mt. Merapi.

Although both communities live on the slopes of active volcanoes, Kemiren village (Merapi) and Pandansari village (Kelud) face distinct vulnerabilities and challenges (see Figure 1). In addition to differences in geographical characteristics, their contrasting historical experiences shape how each community respond to disasters. The eruptions of Mount Merapi, which affect Kemiren, tend to be more dynamic and frequent than those of Mount Kelud. As a result, disaster mitigation and response strategies in Kemiren are more diverse and developed compared to those in Pandansari.

The community in Kemiren is familiar with the *Sister Village* programme’s temporary evacuation initiative that involves relocating to neighbouring villages considered safer from the impacts of volcanic eruptions. These host locations are typically selected based on family ties and have been established for many years. The evacuation includes not only people but also livestock, which are moved to designated communal pens. Although the livestock relocation scheme has become increasingly organised, many residents remain hesitant to return to farming. The sharp decline in livestock prices during previous eruptions has left a lasting impact on those who once relied on livestock farming as their primary source of livelihood. As one informant from Kemiren village stated:

‘When an eruption happens, the price of cattle can drop as badly as goats, and goats can drop just like chickens.’ (Interview with Mr. Pur, Age 55 years old, Kemiren village, 07 July 2023)

This is quite different from the situation in Pandansari village, where a collective livestock evacuation system, such as the communal pens used in Kemiren, has not yet been established. The 2014 eruption was the most severe event experienced by the current generation in Pandansari. As a result, evacuation planning and mitigation strategies have not been developed as thoroughly or systematically as in Kemiren. However, despite the lack of a formal livestock evacuation scheme, most residents in Pandansari continue to work as livestock farmers.

**FIGURE 2:**
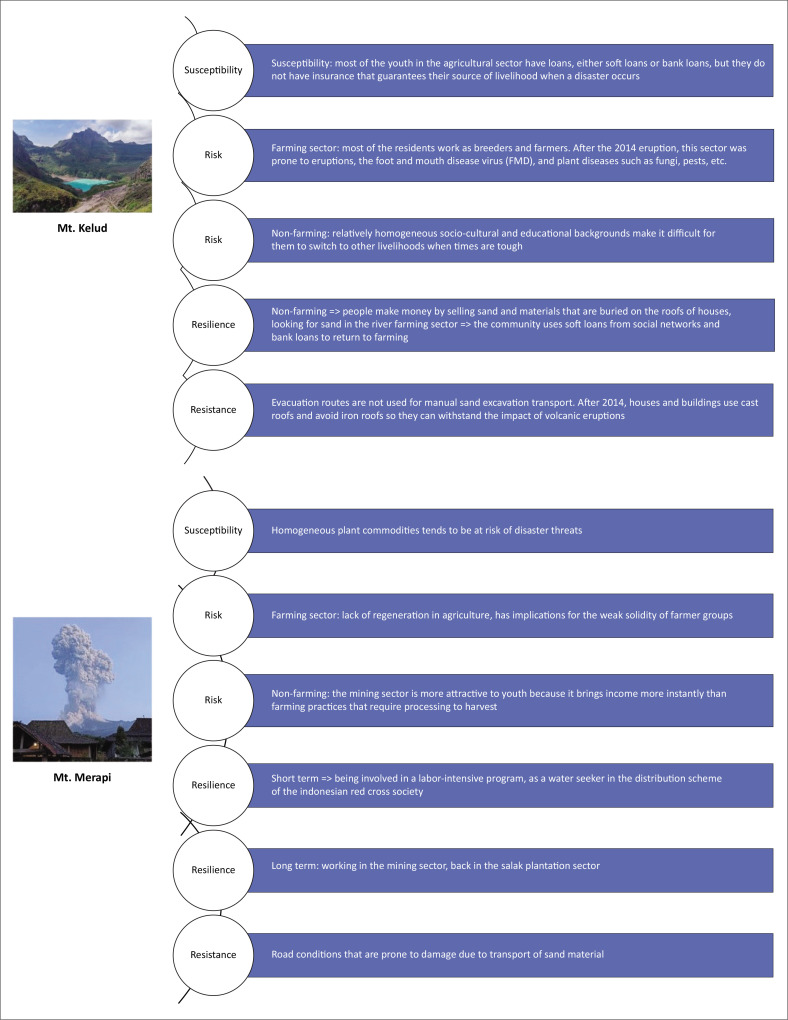
Livelihoods of Pandansari villagers.

The agriculture and livestock sectors remain the economic foundation of the community in Pandansari after the eruption. These sectors are considered key assets that reflect the community’s high resilience (see Figure 2). However, while they serve as the primary source of livelihood for the majority of residents, they are also exposed to risks, particularly those associated with borrowing, including both soft loans and bank loans. As expressed by one informant:

‘People here used to not believe that this village could be affected by an eruption. They still [*thought*] it was safe because Mount Amping is higher up. And before, when Kelud erupted, it never reached us – the wind always blew towards Blitar or Kediri. But after the 2014 eruption, people started to realise that this village could actually be affected by Mount Kelud. Since then, they’ve become more cooperative in all kinds of disaster mitigation, simulations – even things outside of disaster aspect – it’s all become easier to coordinate.’ (Interview with Mr. Parno, Age 58 years old, Pandansari village, 11 May 2023)

The preceding interview excerpt reflects one instance of the residents’ collective memory regarding the changing trajectory of Mount Kelud’s impact before and after the 2014 eruption. Until that point, villagers in Pandansari had relied heavily on local knowledge and shared memory that had guided their response to previous eruptions over the years (Suarmika et al. 2022). Based on their lived experience, volcanic material had almost always travelled towards Blitar and Kediri before 2014. Many believed that Mount Amping, which is higher in elevation than Mount Kelud, acted as a natural barrier during eruptions. This belief reinforced the local myth that Pandansari village was safer than other areas. At the time, village officials, who serve as the most immediate representation of governmental authority, struggled to shift this perception (Antlöv, Wetterberg & Dharmawan 2016). Although residents were willing to participate in mitigation efforts organised by the village government, BPBD, TNI and POLRI, and actively participated in awareness campaigns and evacuation simulations, they continued to hold firm to the belief that their area remained safe, as it had been in the past.

Sulis, a farmer regarded as successful by the local community, shared that he was able to recover after the eruption with the help of a loan from an agricultural equipment dealer he regularly worked with. Although he still suffered losses estimated at up to 500 million rupiah, he chose to take the risk of borrowing from Umi, a local agricultural shopkeeper, rather than from a bank. His decision was based on the mutual trust and motivation that had developed between them. The loan scheme was flexible, as it could be issued in the form of goods, tools, agricultural supplies or money. However, not all farmers have access to such arrangements. Patrons like Umi assess a farmer’s track record, including their work ethic and planting strategies, which influence the perceived risk and potential for profit or loss from planting to harvest.

Other farmers, such as Deni, the Chair of the Farmer Group Association (GAPOKTAN), prefer bank loans. These are often disbursed more quickly and are seen to boost motivation and work ethic because of the strict obligation to repay immediately. As stated:

‘Having a loan gives us farmers a kind of pressure to pay it back, so we work harder to make sure we can repay the debt. Taking out a bank loan is the fastest way to get capital and keep the farming going. But paying it back is also a heavy burden for us. For small farmers like us, there’s really no other option.’ (Interview with Mr. Deni, Age 51 years old, GAPOKTAN, 10 May 2023)

The communities of Kemiren and Pandansari each have their own approaches to survival. In Kemiren, families tend to focus on a single type of work, although job diversification within households is still possible. For instance, a husband and wife may work together in the salak plantation, while their children are employed in the service sector. Intercropping practices are rarely seen in this village, as salak cultivation typically requires full land usage. In contrast, households in Pandansari village tend to follow a shared livelihood pattern that reflects intergenerational knowledge transmission. In a typical household, both husband and wife begin their day by milking cows at dawn until around 07:00 local time (UTC + 7), before heading to the rice fields where they practise intercropping. In the afternoon, they return to the fields with their children and later continue a second round of milking before delivering the milk to the local cooperative. As stated by an informant:

‘Here, husbands and wives usually work together to make a living. It means we help each other to meet our daily needs. We do the farming, and the livestock work as a team. In the morning, we go to the fields, and in the afternoon, we head to the cow shed to do the milking. That’s just how life is for every family in this village.’ (Interview with Mr. Sulis, Age 48 years old, Pandansari Village, 15 May 2023)

Homogeneous livelihoods can become a weakness when communities face threats or disasters. However, livelihood diversification between agriculture and livestock can serve as mutual support, allowing one sector to compensate when the other is affected. For example, when the Foot and Mouth Disease (FMD) outbreak killed nearly 300 cattle belonging to residents of Pandansari, they reported being able to survive by relying on the agricultural sector. Conversely, in times of crop failure, they depend on income from dairy farming to meet their needs.

The experiences of these two villages illustrate that residents understand vulnerability as stemming not only from the threat of volcanic eruptions but also from broader factors beyond natural hazards. Uniquely, efforts to build resilience and resistance are rooted in the collective memory of past disasters. In Kemiren, these efforts are community-led and often subtle. Some local actors have begun to create alternative sectors aimed at drawing young people away from the sand mining industry, even though such change takes time. For instance, Mr Bashori has initiated a goat farm specialising in Sanen and Saphera breeds, with the milk supplied to local factories. Village-owned enterprises (BUMDes) have also introduced certified farming initiatives, which some Kemiren youth are now pioneering despite the dominant employment trend in the sand mining sector (Duha and & Haniek 2023). As stated:

‘It’s true that a lot of young people are more interested in working in the sand mines, mostly because there’s a guaranteed income every day. But there are also many here who are trying their luck in farming, especially through the certified farming programmes run by the village enterprise, BUMDes. For us, many young people feel that mining just damages nature, while farming is a way to take better care of it …’ (Interview with Mr. Sulis, Age 48 years old, Kemiren village, 15 May 2023)

Meanwhile, in Pandansari village, the memory of the devastating Kelud eruption in their village became a turning point of awareness to seek networks and build trust with economic actors outside their area as a precautionary measure if, at any time, their livelihoods were affected again by the Kelud eruption.

### Re-assembling co-production of knowledge as social action after eruption

Co-production has emerged as a counter-discourse to the shortcomings of public (centralised) and private (market-based) service delivery in addressing community needs (Adams & Boateng 2018). When it first gained attention in the United States of America in the 1990s, co-production was often criticised for focusing too heavily on development design and programme planning, rather than on building multi-stakeholder relationships with local communities in the monitoring and implementation stages (Mitlin & Bartlett 2018). Over time, however, the concept has evolved to describe models of shared responsibility designed to enhance the effectiveness of public service delivery. Research conducted by Suyadnya et al. (2022) examined how shared knowledge is formed and manifested in behavioural change as an outcome of multi-stakeholder collaboration in efforts to improve sanitation and clean water services in Pasuruan district, East Java.

The form of co-production examined in this study differs from that discussed in previous research, which often focuses on physical programmes. In this case, co-production refers to the formation of shared knowledge, as manifested in social action, to respond to vulnerability and support post-eruption mitigation. The differing characteristics of the eruptions of Mount Kelud and Mount Merapi have led to distinct impacts and collective memories that shape how communities live in the aftermath. This article argues that co-production is not limited to the pursuit of improved public service delivery. The concept can also be applied to explain how grassroots communities attempt to restore a conducive environment following a crisis. A conducive situation here refers to the ability of communities to regain access to public goods and sources of livelihood, as they had before the eruption.

According to Joshi and Moore (2004), co-production is not solely about cooperation and direct engagement between public agents and civil society in the delivery of services. Communities are composed of individuals from diverse socio-cultural and economic backgrounds. To date, knowledge co-production has frequently been linked to behaviour driven by improved access to public services (Redman et al. 2021; Suyadnya et al. 2022). This article seeks to offer a new perspective by examining how co-produced knowledge developed across different layers of society in areas prone to volcanic disasters influences their social actions in disaster mitigation.

Referring to the Weberian theory, social action can be categorised into four types based on the underlying motivation: traditional, affective, value-rational and instrumental-rational actions (Penta, Wachtendorf & Nelan 2020). Traditional action refers to routine responses or customary ways of doing things. Emotional impulses drive affective action. Value-rational action is guided by deeply held values, in contrast to instrumental-rational action, which is oriented towards achieving specific outcomes (Penta et al. 2020).

This article examines case studies from two villages, Kemiren and Pandansari, through the lens of knowledge dimensions: historical knowledge, mitigation knowledge and action-oriented knowledge. Historical knowledge refers to collective memory regarding past disasters and their periodic recurrence. When individuals lack sufficient knowledge about hazards, they tend to rely on managerial authorities to assess risks and benefits. Conversely, when individuals possess adequate hazard-related knowledge, they are less dependent on such authorities (White, Kates & Burton 2001). However, the situation in Pandansari village was initially different. The community firmly adhered to local beliefs, particularly geomythology surrounding Mount Amping, which they believed would shield them from the material effects of Mount Kelud’s eruptions. The 2014 eruption marked a turning point in this perception, prompting residents to reassess their understanding of disaster dynamics and seek updated knowledge from official authorities (see Table 1).

**TABLE 1:** Dimensions of knowledge in Merapi and Kelud slope communities.

Casuistic	Dimensions of knowledge		
	Historical knowledge	Mitigation knowledge	Knowledge to act
(Kemiren) Merapi	Myths about natural signs and certain practices to be done when these signs appear	Joining *Sister Village* scheme for evacuation mobilisation	The older generation holds *mujahadah* to ask for safety from the danger of eruption and mining threats
(Pandansari) Kelud	Geomythology about Mount Amping protects the village from the materials of Mount Kelud when it erupts. This knowledge makes them feel confident that Pandansari village is safe from the impact of the eruption	Do not wear headgear during evacuation. Participate in socialisation and simulations for the evacuation process. Reconstruct the roof of the house with a dak model after the eruption	Cooperative with the government, building trust and networks with capital owners outside Ngantang sub-district as a precaution for recovery in the event of an eruption

Communities in Kemiren village have faced multiple layers of vulnerability since the 2010 eruption. The closure of the Bebeng River because of volcanic material necessitated normalisation efforts to clear the debris. Over time, these normalisation activities became an entry point for sand mining operations involving heavy machinery. Initially, residents’ concerns were focused on the eruption cycle of Mount Merapi. However, more recently, their fears have shifted towards the risks posed by intensive mining activities.

Our field observations coincided with the emission of a pyroclastic cloud from Mount Merapi on 11 March 2023. On that day, continued to operate along the designated evacuation route. This occurred even though Mount Merapi was at Alert Level 3, with pyroclastic flows reaching up to 1 700 m towards the southwest, precisely the direction in which Kemiren village is located.

The intensity of sand mining activities in Kemiren has implications that extend beyond the physical deterioration of infrastructure, including damage to evacuation routes and the impact of vibration on roadside houses and buildings. At a micro level, this activity also appears to generate friction between the older and younger generations. The older generation struggles to pass down values and knowledge related to traditional livelihood practices in agriculture and plantation sectors, as younger people are increasingly drawn to the sand mining industry because of its promise of quicker income. At the same time, those engaged in the sand mining sector also contribute to local infrastructure, particularly in supporting village primary schools. For instance, when the vibration from heavily loaded trucks causes damage, such as broken windowpanes, the school committee can submit funding proposals to parents, including those working in the mining industry. This arrangement illustrates how power and influence operate within community relations (Anderson 2005). Islamadi, a head of Sambirejo Hamlet in Pandansari village, reflected on a different kind of hardship:

‘As heavy as the eruption was, the FMD outbreak has been even harder. During the eruption, I didn’t worry about money. I used to earn two million, but now I’m down to three hundred thousand, and the bank hasn’t even come to collect yet.’ (Interview with Mr. Islamadi, Age 45 years old, Pandansari village, 20 May 2023)

These findings suggest that natural hazards do not solely drive vulnerability and resistance in disaster-prone communities. On the contrary, they are often shaped by social reproduction, economic processes and forms of exclusion. A volcanic eruption is typically perceived as a generalised disaster affecting all members of a community. In contrast, crop failure, livestock disease or the negative effects of mining are seen as issues that affect only specific individuals. This differentiation highlights how social exclusion, both conscious and unconscious, can emerge within a single community.

### *Mujahadah*: Seeing ritual from co-production of knowledge about ecological safety

In mid-March 2023, while Mount Merapi remained at Alert Level III, dozens of sand trucks continued to operate along the damaged evacuation route of Kemiren village, despite the presence of potholes. At the same time, caretakers, elders and members of the older generation gathered at night to perform the *mujahaddah* ritual, seeking protection and safety. Informants in Kemiren village did not frequently mention the term *mujahaddah*. Still, it was mentioned several times by village elders and older residents, whose livelihoods depend on farming and plantation work.

According to Mbah Parto, a respected elder of Kemiren village whom we interviewed, *mujahaddah* is a form of *tirakat* involving overnight *dhikr* (chanting or remembrance) held in an open field, with the hope that the prayers will reach God. Traditionally, *mujahaddah* was practised as an expression of submission to the uncontrollable forces of Mount Merapi during times of volcanic unrest (Ricklefs 2012). Today, however, the ritual is performed not only in response to the threat of eruption but also as a spiritual plea against the growing impact of large-scale sand mining.

Volcanic eruptions consistently leave a dual impact on communities: destruction on the one hand, and increased soil fertility on the other. The destructive effects are often short-lived, as labour-intensive recovery programmes enable residents to work collectively to restore damaged infrastructure and rehabilitate salak plantations. The eruption of Mount Merapi also disrupted the flow of the Gendol River, causing it to be completely flattened. Under the pretext of river normalisation, sand mining activities began, and have continued ever since, now extending even to the slopes of Merapi. This ongoing extraction has triggered a collective response from the community in the form of the *mujahaddah* ritual. It has become a symbolic act of final surrender, performed when residents feel they can no longer voice their grievances through formal governmental channels.

According to Mr Suhono, *mujahaddah*, as a means of anticipating the eruption of Mount Merapi, is conducted on a rotational basis in each neighbourhood unit (RT) in Kemiren village, following an agreed schedule. The ritual takes place every Friday eve (Thursday night) after the *Isha* prayer (Ricklefs 2012). The activity typically involves the communal recitation of tahlil, led in turn by local residents. Beyond its spiritual purpose, *mujahaddah* also serves as a space for communication and social interaction, enabling residents to share information, voice their concerns and collaborate to find solutions. *Mujahaddah* has been practised for generations, passed down by the ancestors. Given that Mount Merapi has been erupting since at least 1930, the tradition of *mujahaddah* has long been part of the community’s cultural response to living in a disaster-prone environment (Ricklefs 2012).

The eruption of Mount Merapi resulted in the deposition of abundant volcanic material, particularly sand, which investors have exploited through ongoing mining activities. Over time, the meaning of *mujahaddah* for the villagers of Kemiren has shifted. While it was initially performed as a spiritual plea for protection from eruptions, it is now also used to express collective hope that the ongoing mining activities will cease.

According to Mbah Parto, a respected elder in Kemiren village, unregulated and continuous mining poses a serious threat to the local ecology. As translated from Javanese:

‘In the end, the area around Mount Merapi will face drought. Forget farming … there won’t even be water to drink. Mount Merapi will collapse, seawater will rise, and people will have to use gethek boats to get around. Mount Merapi is the buffer for Java. If it’s dredged too much, it’ll vanish into the ocean. Merapi is the partner of the South Sea … it’ll all turn into a swamp.’ (Interview with Mbah Parto, Age 75 years old, Kemiren village, 16 March 2023)

Social construction theory refers to three key aspects: externalisation, objectification and internalisation (Ertanto, Eskasasnanda & Uzma 2022). *Mujahaddah* is an essential element of the community’s response to the threats posed by both the eruption of Mount Merapi and ongoing mining activities. This ritual, performed routinely and passed down across generations, represents a significant part of the externalisation process, where individuals begin integrating themselves into the community through shared religious practices (Schlehe 2017). The unpredictable threat of Mount Merapi, combined with lived experiences of past eruptions, contributes to the formation of a collective memory around the volcano’s dangers. Conventional preparedness efforts, such as community training to recognise warning signs or implement disaster mitigation strategies, have not been perceived as sufficient to protect residents from the eruption’s impact. As a result, *mujahaddah* has come to represent a form of spiritual resilience, where the community turns to divine protection as their primary safeguard, especially in light of increasingly intensive mining activities on the slopes of Merapi (Schefold 2013).

**FIGURE 3:**
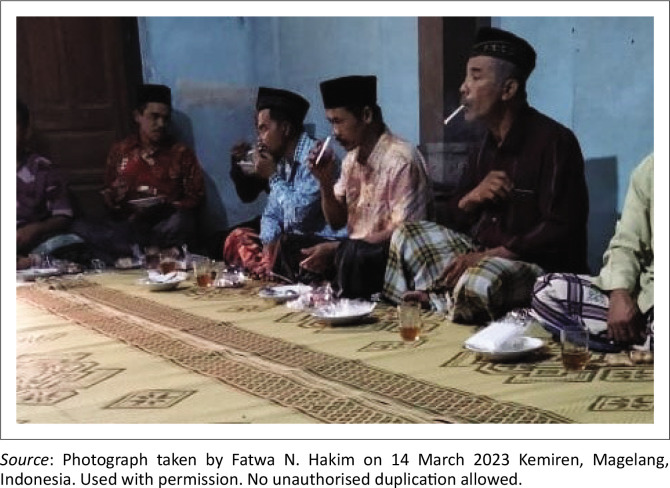
*Mujahaddah* ritual in Mt. Merapi community.

The process of objectivation is reflected in how individuals begin to understand *mujahaddah*, internalise it within themselves and transmit it to others, particularly the younger generation. This stage is vital to the functioning of social construction within a society (Zittoun & Gillespie 2015). *Mujahadah* (see Figure 3) has been practised for generations as a form of religious heritage. It is passed down as both a guideline and a way of life, enabling people to draw lessons and wisdom from ancestral traditions. At this point, the process of internalisation takes place, wherein religious rituals become an integral part of community identity. The community comes to understand *mujahaddah* not merely as a religious act, but as a meaningful effort to resist and cope with the disaster posed by the eruption of Mount Merapi.

In Kemiren village, *mujahaddah* has taken on two distinct forms, reflecting different motivations within the community. The first group performs *mujahaddah* with the hope of being protected from the dangers of Mount Merapi’s eruptions and in the hope that sand mining activities will cease, or at least that the harmful consequences of mining can be avoided. The second group, in contrast, performs *mujahaddah* so that sand mining can continue smoothly and safely. This second group primarily consists of individuals involved in mining activities, particularly those residing in the village who are directly affected by the conditions of Merapi. These differing motivations reveal how people interpret the ecological function of religion in varied ways, shaping their requests through ritual. *Mujahaddah* is regarded both as a spiritual plea for protection from disaster and as a form of community-based disaster awareness, fostering resilience (Yasinatul et al. 2022).

The mujahadah ritual performed by sand mining workers in Kemiren village is held at night near the mining sites. Following the eruption of Mount Merapi, sand mining activities increased significantly because of the abundance of volcanic material. Even when Merapi was emitting incandescent lava, mining continued. This has had a considerable impact on the social dynamics of Kemiren village. *Mujahaddah*, in this context, serves as a form of spiritual reinforcement in the face of the community’s evolving conditions and challenges. The younger generation has increasingly entered the mining sector, viewing it as a livelihood that offers greater economic returns. For many, sand mining became a primary source of income after the Merapi eruption. It enables families to meet their daily needs, and for those involved in mining, *mujahaddah* is performed as a request for safety and protection during their work.

This divergence in the meaning of *mujahaddah* reflects the complex social landscape in Kemiren. On one hand, it is practised to seek safety from the threat of Merapi’s eruptions; on the other, it is invoked to ensure safety during mining activities. Sand mining workers are especially attentive to the environment near Mount Merapi, which they regard as part of their ancestral heritage (Schwartz-Marin et al. 2022). Their belief in the land as a source of food and livelihood reinforces their connection to the environment. At the same time, this rationale has sparked criticism and opposition from other members of the community who view the mining activities as ecologically damaging. These tensions have also contributed to changes in the community’s patterns of communication. Previously, information and warnings were typically conveyed by elders or traditional leaders to the rest of the community, but this mode of dissemination is beginning to shift.

### Building trust: Building co-production of knowledge about resilience in Pandansari village

**FIGURE 4:**
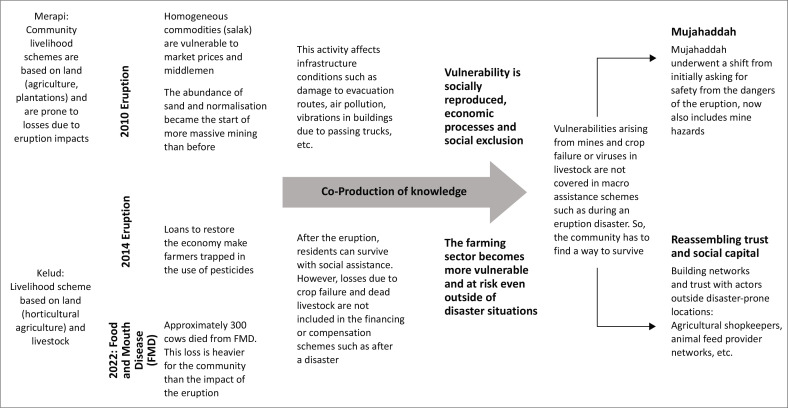
Process co-production knowledge community in Mt. Merapi and Mt. Kelud.

Co-produced knowledge plays a vital role in building resilience through the development of trust (see Figure 4). Figure 4 illustrates the challenges faced by communities affected by natural disasters and highlights the importance of collaborative strategies and support networks in enhancing social resilience in disaster-prone areas. For communities living alongside the constant threat of volcanic eruptions, environmental conditions have a profound impact on their livelihood systems. The 2014 eruption of Mount Kelud resulted in severe losses in key livelihood sectors, including agriculture and animal husbandry. Livelihood systems that depend heavily on the natural environment surrounding Kelud are inherently more vulnerable than those linked to economic activities outside disaster-prone zones (Saparita et al. 2024). Following the 2014 eruption, a growing awareness emerged of the importance of building trust and forming connections with stakeholders beyond the immediate disaster area (Horwell et al. 2019; Sudarmanto 2020).

Farmers in the region are generally divided into two groups, older and younger, with differences in age and experience shaping their preferences for trust-building strategies. Younger farmers tend to rely more on bank loans, agricultural supply shops or middlemen. One story that stands out is that of Pak Sulis, a young farmer widely regarded as someone who successfully rebuilt his livelihood after experiencing severe loss.

The 2014 eruption left him with damage exceeding 600 million rupiah. Still, he managed to recover after receiving support from Umi, a seller of farm equipment and agricultural supplies based in Pujon, Batu City. The loan was not based on formal collateral, but on Sulis’s proven work ethic and the relationship he had built with Umi. According to her, his crop failure was not the result of poor farming practices, but rather the impact of an external disaster. As a result, she was willing to provide him with the necessary support, particularly in the form of medicines, seeds and farming equipment. As shared:

‘Yeah, we really suffered losses because of the eruption, and to cover those losses and get some capital again, we had no choice but to take a loan from the bank. In a tough situation like that, after the eruption, there really wasn’t any other option.’ (Interview with Mr. Sulis, Age 48 years old, Kemiren village, 15 May 2023)

Other young farmers choose to seek capital from banks and rent land owned by others, as Deny, an onion farmer, does. On average, young people cultivate land that belongs to others through rental agreements. Because of limited informal connections, Deny considers banks as a last resort for borrowing capital. According to the village government, most young farmers are more willing to take out bank loans compared to the older generation. This reliance on formal capital has implications for their work ethic, as they feel pressure to work harder to meet monthly loan repayments. To reduce the risk of crop failure, many resort to using pesticides. This approach is common among young farmers with outstanding loans, who often feel compelled to rely heavily on chemical treatments. As horticulturalists, they have tested the difference themselves and find it challenging to trust organic crop labels in supermarkets. According to their experience, if one plot is pesticide-free while an adjacent plot is treated with pesticides, the untreated crops become more vulnerable to pests and disease. As a result, many farmers use small plots of land near their homes to grow pesticide-free crops exclusively for personal consumption. In disaster-prone areas such as Kelud, the pressure of loan repayments and market expectations forces farmers to adopt such strategies as a means of securing their livelihood systems.

**FIGURE 5:**
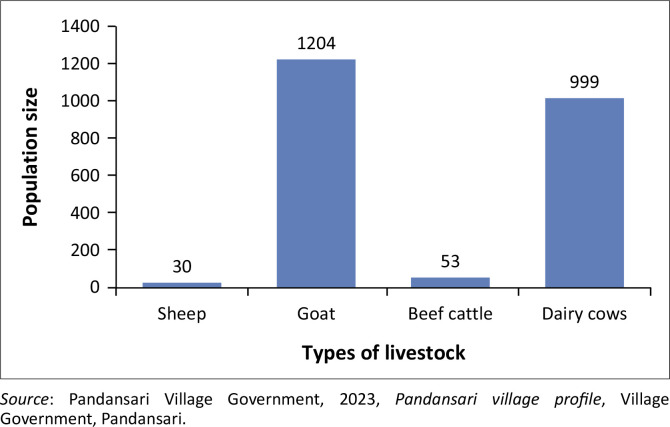
Livestock population in Pandansari village.

Similar to horticultural farmers, cattle farmers in Pandansari village are also highly vulnerable to outbreaks of disease affecting their livestock. The outbreak of FMD in 2022 is a notable example of a disaster unrelated to the eruption that severely impacted the community. As the Head of Pandansari village stated (2023), ‘Diseases can strike at any time. We must be ready to face illnesses affecting both crops and livestock’. Eruption-related disasters and viral outbreaks are treated differently by government authorities. While volcanic eruptions tend to affect all layers of society equally, resulting in broadly distributed aid, FMD only impacts livestock owners. As a result, the compensation and treatment provided are often not proportional to the scale of the overall case reported (Prasetia, Primatika & Nugroho 2025). Amid this uncertainty, farmers primarily relied on informal, word-of-mouth communication and improvised care techniques. They installed fans in their cattle sheds to reduce heat, and administered remedies such as antangin, Pocari Sweat and sugar solutions to their cows. In addition to these emergency treatments, many cattle owners stayed up through the night, driven by the belief in *pageblug* – a Javanese concept referring to a mysterious, widespread plague. They relied on their hearing: if they heard a cow *bengah* (a laboured breathing sound), it signalled that the animal was close to death. Farmers would rush to the source of the noise, often overcome with grief upon discovering that their main source of livelihood had died. In the aftermath, cattle owners not only had to deal with emotional loss but also faced the financial burden of arranging care and burial for the animals. Finding help for the burial was especially difficult, as every farmer was preoccupied with saving their livestock (see Figure 5).

The hamlet in Pandansari village with the highest number of cattle deaths is Kutut. Situated at one of the highest elevations in the village, Kutut is home to several young farmers who are considered successful by the local community, some of whom own more than 100 cows. These young farmers typically have not completed formal education, as formal schooling is not regarded as a benchmark of success in the village. In Pandansari, success is defined by one’s work ethic and resilience in the face of crisis. Those who suffered significant losses during the eruption but managed to rebuild and continue their businesses are held in high esteem. Moreover, local notions of welfare include the ability to provide employment opportunities within the community. A person who can absorb local labour is seen as demonstrating social responsibility and care for others:

‘The person who owns this livestock did not graduate from school, only up to grade 4, but he has a lot of money, school also does not guarantee that he can have a lot of money, the important thing is field school.’ (Interview with Mr. Islamadi, 10 May 2023)

**FIGURE 6:**
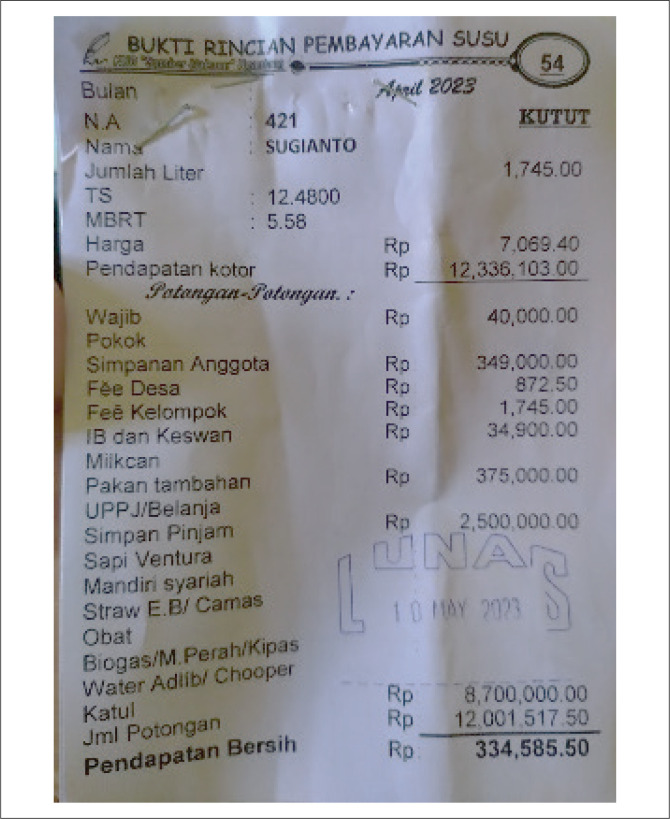
Receipt for milk payment details and cooperative member finances.

In crisis situations, farmers in Pandansari commonly rely on credit loans from local cooperatives (see Figure 6). Meanwhile, larger-scale farmers or those with extensive livestock operations often utilise wider networks beyond Malang City to secure supplies of animal feed. For example, they may coordinate with maize farmers in Jombang District (Muwakhid, Kalsum & Wajdi 2023). Since the eruption, it became clear that meeting animal feed needs would be a major challenge, as volcanic ash had contaminated much of the surrounding vegetation traditionally used for feed. In response, large-scale farmers began to establish relationships and maintain communication with farmers outside Malang District to ensure continuity of supply in future crises. Small-scale farmers, in contrast, typically rely on large-scale farmers to submit requests on their behalf when feed shortages occur. For these farming communities, building trust and social capital is not solely a means to access financial resources. In critical situations, the ability to obtain animal feed is an urgent, non-material need that cannot be delayed or substituted, making strong social ties essential for survival in a critical situation.

## Conclusion

The same disaster can lead to different recovery trajectories, influenced by the interplay of social, economic and environmental factors. Communities must navigate these complexities, often relying on a combination of past experiences and adaptive strategies to rebuild and thrive in the aftermath. Systematically addressing vulnerabilities enhances preparedness for future challenges and supports resilience, not only against immediate threats but also in the face of long-term socio-economic shifts. The socio-cultural characteristics of a community influence how they respond and how they build resilience and shared knowledge as forms of collective action, particularly in contexts where government intervention is absent.

Based on the findings, few residents in Pandansari village anticipated that the 2014 eruption of Mount Kelud would have such a devastating impact, as previous eruptions had largely affected villages in other districts. In contrast, residents of Kemiren village had long incorporated volcanic activity into their social practices. Their fear had shifted from the eruption itself to the growing threat posed by sand mining as a consequence of Merapi’s volcanic activity. The differing contexts of the same disaster led to the development of distinct co-produced disaster mitigation strategies. In Kemiren, the lack of clear avenues for addressing the negative impacts of sand mining led residents to express submission through religious ritual. *Mujahaddah* was performed as a plea for safety from the cumulative threats of both volcanic activity and ecological degradation. On the other hand, pro-mining groups also engaged in *mujahaddah* but their aim was to secure protection for their mining activities. This dual use of the same religious ritual illustrates how religion can be mobilised for opposing ecological objectives.

In contrast to Kemiren, people in Pandansari village, the majority of whom are farmers and breeders, have increasingly recognised the importance of building trust with actors beyond the immediate disaster zone. They seek to establish connections with capital owners and external economic stakeholders to buffer against losses caused by eruptions. This study finds that the role of government and aid providers tends to be moment-driven and evidence-based: their presence and support are most visible during the official disaster period. Once the status of a disaster subsides, communities are often left to navigate subsequent crises independently. The Pandansari community’s response to the FMD outbreak, farmers’ pursuit of capital loans to cope with crop failure and the older generation in Kemiren’s turn to *mujahaddah* in response to mining-related disruptions are all examples of co-production taking place in crisis contexts outside officially recognised disaster events. These cases reveal the dual role of official disaster relief: as both a temporary solution and a trigger for the oversimplification of new vulnerabilities that arise outside the standard disaster framework. While all eruption victims receive support during a recognised disaster, those affected by FMD, crop failures or the impacts of sand mining often experience these crises without comparable assistance. This results in fragmented and partial co-production efforts that are difficult to unify across the entire community.

The limitations of this research offer opportunities for future study. Firstly, data limitations may have affected the depth of analysis regarding the socio-economic context, relevant research outputs and the long-term impacts post-eruption, particularly in comparing the Merapi and Kelud communities. Secondly, variability in community responses makes it difficult to generalise findings across different locations. Thirdly, the narrow focus on agriculture and livestock may have overlooked other sectors impacted by the eruptions, such as local industry and tourism. Finally, sustained community engagement in co-produced knowledge may not be fully realised, especially where distrust towards external actors or government institutions persists.

### Areas for further studies

This research highlights two key areas with potential for future development:

- Firstly, the integration of multidisciplinary approaches, combining economic, social and environmental perspectives, to achieve a more holistic understanding of disaster resilience.
- Secondly, the development of crisis response models, particularly those that are effective and accompanied by recovery roadmaps, which integrate both local knowledge and scientific expertise for improved risk management.

By addressing these limitations and focusing on broader research potential, future studies may contribute to strengthening community capacity in anticipating and mitigating the impacts of volcanic eruptions.
